# *Ficus Carica *L*.* Latex: Possible Chemo-Preventive, Apoptotic Activity and Safety Assessment

**DOI:** 10.22037/ijpr.2020.1101151

**Published:** 2020

**Authors:** Fereshteh Jeivad, Nargues Yassa, Seyed Nasser Ostad, Zahra Hassannejad, Gholamreza Hassanzadeh Gheshlaghi, Omid Sabzevari

**Affiliations:** a *Department of Toxicology and Pharmacology, Faculty of Pharmacy, Tehran University of Medical Sciences, Tehran, Iran. *; b *Drug Design and Discovery Research Centre, Tehran University of Medical Sciences, Tehran, Iran. *; c *Department of Pharmacognosy, Faculty of Pharmacy, Tehran University of Medical Sciences, Tehran, Iran. *; d *Toxicology and Poisoning Research Centre, Tehran University of Medical Sciences, Tehran, Iran. *; e *Pediatric Urology Research Center, Section of Tissue Engineering and Stem Cells Therapy, Children’s Hospital Medical Center, Tehran University of Medical Sciences, Tehran, Iran.*; f *Department of Anatomy, School of Medicine, Tehran University of Medical Sciences, Tehran, Iran.*

**Keywords:** HCC, Cytotoxicity, LD50, Phytochemical analyses, Lupeol

## Abstract

Hepatocellular carcinoma is the third cause of cancer-related mortality with the low 5-year survival in which more than 50 percent of patients have recurrent cancer within 2 years of treatment. The present study investigated the cytotoxicity and lethal dose of *Ficus carica *L. (Figure) latex and phytochemical composition of effective fraction. Figure latex was collected in summer and 4 fractions of Figure latex were prepared. The cytotoxic effect of each fraction was studied and the most effective fraction was selected for apoptosis assay, acute toxicity study, and phytochemical analysis using column chromatography. The isolated compounds were identified by ^1^H-NMR, ^13^C-NMR, and mass spectroscopy. Chloroform fraction was the most effective fraction with the IC_50_ value of 0.219 and 0.748 mg/mL for HepG2 and NIH cell lines, respectively. Presence of cells in early apoptotic phase was documented by flow cytometry assay. Single dose administration of 2g/kg of fraction did not cause any death. Phytochemical analyses confirmed presence of lupeol acetate and lupeol palmitate in chloroform fraction**.** The present study revealed that the chloroform fraction is not only 3.4 times more toxic in HepG2 cell line but also has low *in-vivo* toxicity which could be considered as a good candidate for a chemo-preventive agent.

## Introduction

Hepatocellular carcinoma (HCC) is one of the causes of cancer-related death worldwide and is the fourth common cancer in men as well as seventh in women. HCC is considered as a global concern with 0.5 million new cases each year which shows a trend of dramatic increased (1, 2). HCC is the fastest cause of cancer-related death in the USA due to its low 5-year survival (3). Current available treatments of HCC are not efficient enough due to complicated pathogenesis and molecular pathway of the cancer. Despite various therapeutic options, 50 percent of the patients have recurrent disease during two years (4). 

Medicinal plants and their compounds have gained therapeutic consideration as a result of their multi-level and target interactive beneficial effects (5). In between, Ficus carica (FIg) is belong to Moraceae family which is a delicious fruit as well as valuable medicinal plant which grows up in Mediterranean area (6). Various parts of the plant including fruit, leaves, root, and latex have been traditionally used in medicine (7). Anti-papillomatosis (8), anti-inflammatory (9), anti-angiogenesis (10, 11) and anthelmintic activity (12) were reported for the Fig latex. Cytotoxic effect of Fig latex polar fraction has been reported by various studies (11, 13-18). Moreover, a study by Tezcan *et al. *demonstrated no cytotoxicity when the non-polar fractions of latex were used (6). Fig latex is myriad of compounds and its phytochemical study confirmed presence of β-sitosterol, palmitoyl, linoleyl, stearyl, and oleyl (19). Another study reported presence of triterpenoid compounds such as α-amyrin, β-amyrin, lupeol, β-sitosterol, and stigmasterol in the latex (20) which have high molecular weights and low water solubility (21). These features may lead to poor solubility of these compounds in culture media and low delivery to cells, which resulted to no cytotoxicity.

Liposomal delivery is an appropriate delivery system to overcome triterpenoids solubility and delivery limitations. In this content, Henry *et al*. used a liposomal delivery system of terpenoid compounds for cosmetic use (22). The use of phosphatidylcholine in complex with hydrophilic and large molecular weight compounds (called phytosome) has been reported (23, 24) but there is no available data regarding the solubility of hydrophobic plant compounds in culture media. 

The first aim of the present study was to improve solubility of two non-polar fractions of the Fig latex for cellular delivery using the liposomal delivery system. The second aim was to study cytotoxic effect of Fig latex fractions on HepG2 and NIH cell lines to choose the most effective fraction. The third aim was phytochemical analysis, lethal dose evaluation and apoptotic activity of the most effective fraction.

## Experimental

*Latex and extraction*

**Figure 1:**
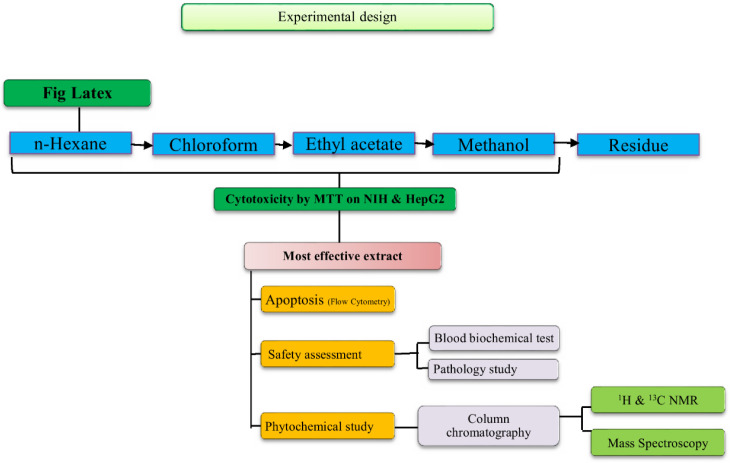
**Experimental design of study**

*F carica* latex was collected drop by drop in the north of Iran (Sari, Mazandaran Province) through picking the green fruit in July 2015 and then the collected latex was stored at C -20. Extraction of Fig latex was performed using 4 solvents including n-hexane, chloroform, ethyl acetate, and methanol (Merck, Germany), respectively (25). The experimental design was summarized in Figure 1.

*Extract preparation for cell culture media*

The methanol and ethyl acetate fractions were dissolved in cell culture media but suitable solvent was not available for the non-polar fractions. Therefore, the fractions were prepared in the liposomal form using egg yolk phosphatidylcholine (EPC) (Sigma, USA) (26). The dry weight of each fraction was mentioned in Table 1

*Liposome preparation*

Each fraction of n-hexane and chloroform was dissolved in chloroform separately, EPC was added to dissolve fraction in 1.5:1 ratio. The mixture was then dried under vacuum at room temperature till the lipid film is formed. The hydration was performed by adding PBS at 65  C for 2 h with vortexing every 5 min. after that time (27). All procedure was performed in sterile condition.

*Particle size*

Particle size and polydispersity index (PDI) were measured by Photon Correlation Spectroscopy (PCS) using a Zetasizer nano zs (Malvern Instruments Ltd, UK). The samples were dissolved in PBS at 1mg/mL and filtered through 0.22-μm syringe filters. Measurements were carried out in triplicates.

*Cell line and culture*

The HepG2 and NIH3T3 cell lines were provided by Pasture institute, Tehran, Iran. The cells were cultured at 25 mL flask in RPMI 1640 medium (Biosera, USA) containing 10 % Fetal Bovine Serum (Gibco, USA) and 1% pen/strep (PAA, Austria) in saturated humidity and 5% CO_2_ incubator.

*Cytotoxicity assay*

The cytotoxicity of 4 fractions of Fig latex and isolated compounds from the most effective fraction were measured by MTT at 24, 48, and 72 h. For MTT assay, the cells were seeded in 96 well plate, 1× 10^6^ cell per well. The cells were treated 18 h after seeding with various concentrations of fractions. At the end of incubation periods, the RPMI 1640 medium of each well was replaced with MTT (100 of 0.5 mg/mL MTT in RPMI 1640 without FBS) and the plate was incubated for 4 h in saturated humidity and 5% CO_2_ incubator. 100 µL DMSO was added to each well, gently shaken, and the absorbance was read by ELISA at 570 nm (28).

*Cell apoptosis assay*

Cell apoptosis was assessed by flow cytometry. The cells were seeded on 6 well plates and treated with chloroform fraction and EPC at selected concentrations. After 14 h, FITC-labeled Annexin V/PI staining was added according to the manufacturer’s protocol (BD Company, USA). Briefly, 1×10^6 ^cells/well were suspended in buffer containing FITC-conjugated Annexin V/PI. The samples were analyzed by flow cytometry and data for at least 10,000 cells were collected (28). 

*Lethal dose determination*

Lethal dose was determined in female Syrian mice (25-30 g) in six groups consisting of 10 mice in each group. The animals were fast 18 h before study with free access to water. The chloroform liposome fraction was prepared by EPC (1:1.5) and was administered to the animal by intraperitoneal injection (i.p). Control group received an equal dose of EPC (Table 2). Signs of intoxication were assessed for 4 h after administration with 30 min interval. Mortality was measured every 24 h until 96 h and the animals were monitored for 14 days. The animal experiment was performed according to TUMS ethic committee (IR.TUMS.REC.1394.1784) (29).

*Biochemical, macroscopic and microscopic evaluation*

After 14 days, the animals were sacrificed by spinal dislocation and abdominal organs were observed. The blood samples were collected for AST, ALT, and ALP analysis in serum by Elisa kits (Teb Gostaran Hayan, Iran). Liver sections were isolated, fixed in 10% formalin, and stained with Hematoxylin and Eosin dye for histopathology examination. 

*Purification and identification*

Chloroform fraction, as the most effective fraction, was chromatographed on thin layer chromatography (TLC) for spot numbered of this fraction. On TLC, two spots were found to be major components of chloroform fraction and aimed to isolate by column chromatography. 500mg of chloroform fraction was dissolved in chloroform and mixed with silica gel (230-400, mesh) to produce a uniform mixture. The solvent was evaporated at room temperature. The mixture was added to glass column (diam 1× height 60 cm) packed with silica gel (230-400, mesh) and hexane as solvent. The fraction was eluted with a gradient of hexane and chloroform solvents. Following that, the fractions were collected in numbered test tubes and each of them was re-chromatographed on TLC with hexane: chloroform (1:1) solvent system. The fractions with a single spot on TLC were selected for ^1^H, ^13^C, and Mass spectrometry analysis (30).

*Statistics*

The data were analyzed by Graph Pad Prism 5.04. One-way ANOVA and Tukey’s *post hoc* were used for comparison between the groups, the p-value less than 0.05 was considered as significant (31).

## Results

*Particle Size*

The liposome size was measured by DLS and the distribution of the size by number was 97 % in 141 ± 15 nm with PDL index of 0.419.

*Cytotoxicity assay*

Various concentrations (0.6, 1.25, 2.5, 5 and 10 mg/mL) of Fig-latex methanol fraction showed a growth stimulation activity on HepG2 cell line. While ethyl acetate, chloroform, and hexane fractions were cytotoxic on HepG2 cell line, chloroform fraction was the most cytotoxic fraction with the lowest IC50. The IC50 value at 24 h after treatment was 0.219 and 0.748 mg/mLfor HepG2 and NIH cell lines, respectively, Table 3.

*Cell apoptosis assay*

**Figure 2:**
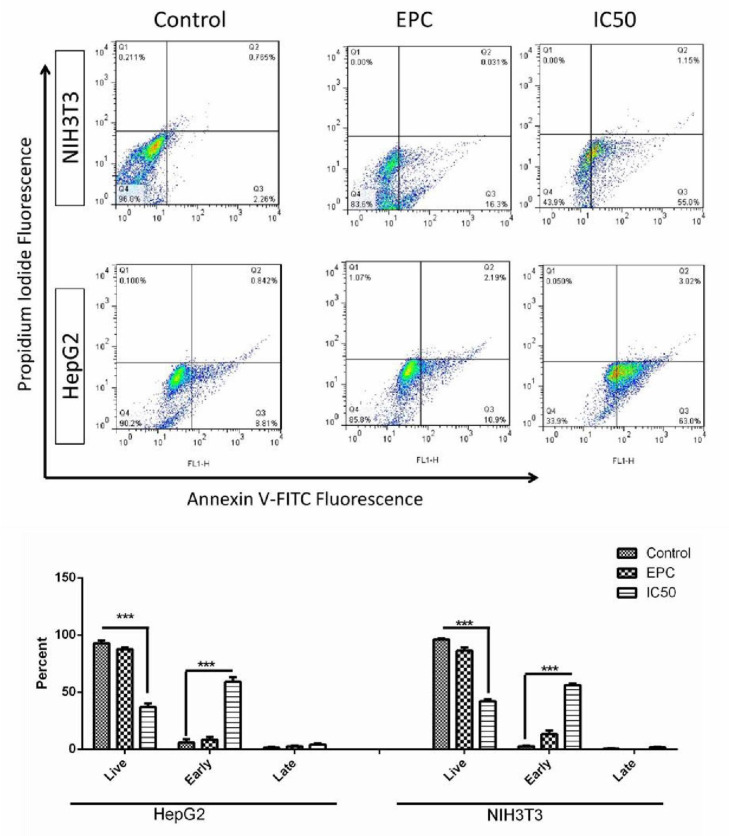
**Cell apoptosis due to chloroform fraction of Fig latex on HepG2 and NIH3T3 cell lines**

Liposomal form of Chloroform fraction of Fig latex induced apoptosis on both NIH3T3 and HepG2 cell lines. According to results shown in Figure 2, the EPC did not increase cell death in comparison to the control.

*Lethal dose determination*

Administration of liposomal form of chloroform fraction caused 3 deaths in 3 g/kg group, while no death was observed following administration of 1 and 2 g/kg. Administration of EPC in 3 dose levels of 1.5, 3, and 4.5 g/kg caused no death in the treated groups.

*Biochemical evaluations*

The liver function tests of treated animals with chloroform fraction of Fig latex and its vehicle (EPC) were shown in Table 4.

*Histopathological assessment*

**Figure 3 a:**
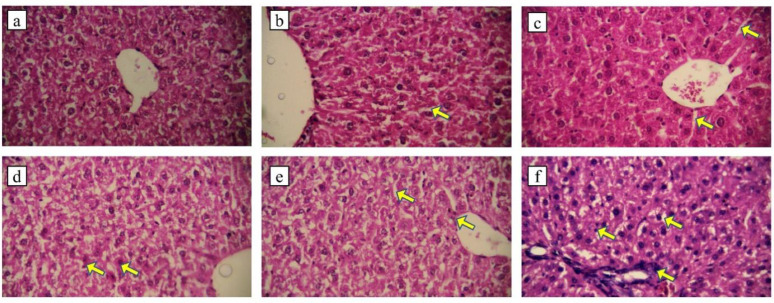
EPC 1.5 g/kg, b: EPC 3 g/kg, the arrow shows dilation of sinusoids. c: EPC 4.5 g/kg, the arrows show dilation of sinusoids. d: fraction (1 g/kg), the arrows show bleeding e: fraction (2 g/kg), the arrows show dilation of sinusoids and kupffer cells. f: fraction (3 g/kg), the arrows show fibroblast accumulation, kupffer cells and sinusoids dilation. (H & E staining, X400).

Administration of EPC (1.5 g/kg) did not cause liver tissue damage with normal hepatocytes and kupffer cells within the sinusoids (Figure 3a). The histology of liver in group b (3 g/kg EPC) was normal but the number of kupffer cells increased compared to group a, (Figure 3b). In group c, the number of kupffer cells increased and the sinusoids were relatively dilated (Figure 3c). The hepatocytes and sinusoids were normal in the animals receiving dose of 1 g/kg chloroform fraction, but in some areas, the bleeding was observed (Figure 3d), the liver histology in group 2g/kg was normal but the kuppfer cells relatively increased and sinusoids were relatively dilated (Figure 3e). Administration of liposomal fraction at dose of 3 g/kg highly increased kupffer cells, fibroblast accumulation and hepatocyte death, in addition, the sinusoids are so dilated due to the treatment (Figure 3f).

*Phytochemical analyses*

**Figure 4:**
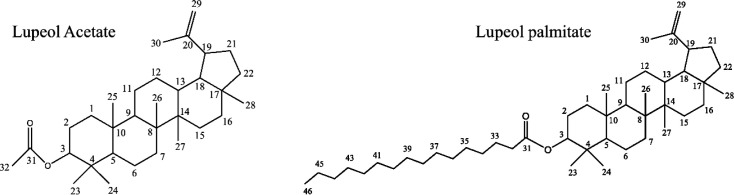
Two isolated compounds from chloroform extract of Fig latex

Phytochemical analysis was performed for chloroform fraction by TLC and column chromatography. The numbers of spots in this fraction were determined by TLC and were separated by column chromatography. Two major compounds were isolated from the chloroform fraction and identified by ^1^H-NMR, ^13^C-NMR, and mass spectroscopy namely lupeol acetate and lupeol palmitate (Figure 4).

Lupeol Acetate; EIMS for C32 H52O2; m/z (rel. Int.): 468[M+] (17.32%), 453 (18.89%), 393(16.53%), 408 (4.72%), 357(3.12%), 218(11.71%). 189 (55.11%), 109 (59.5%), 43(100%).

^1^H-NMR (CDCl3, 500 MHz): δ 4.69(1H, *bs*, H29), 4.57 (1H, *bs*, H29), 4.47((1H, *m*, H3), 2.04 (3H, *s*, H32), 1.68(3H, *s*, H30), 1.03(3H, *s*, H25), 0.94 (3H, *s*, H28), 0.85(3H, *s*, H23), 0.84 (3H, *s*, H24), 0.83 (3H, *s*, H26), 0.79 (3H, *s*, H2). 

^13^C-NMR (CDCl3 125 MHz): δ 171.01 (C31), 117.59 (C20), 109.35 (C29), 80.98 (C3), 55.39 (C5), 50.35 (C9), 48.30 (C18), 48.01(C19), 20.94 (C32).

Lupeol palmitate; EIMS for C46 H80O2; m/z (rel. Int.): 664[M+] (21.87%), 649 (5.46%), 445 (3.12%), 408 (14.06%), 393 (12.5%), 218 (21.87%), 204 (34.37%), 189 (63.28%), 175 (15.62%), 121 (32.03 %), 43 (100%)

1H-NMR (CDCl3, 500 MHz): δ 4.68 (1H, *bs*, H29), 4.57 (1H, *bs*, H29), 4.47 (1H, *dd,* *J *= 11.03, 6.04, H3), 2.04 (3H, *s*, H32), 1.68 (3H, *s*, H30), 1.03(3H, *s*, H25), 0.94 (3H, *s*, H28), 0.85(3H, *s*, H23), 0.84 (6H, *s*, H24, H26), 0.79 (3H, *s*, H27), 0.88(3H, *t*, *J *= 6.05, H46).

^13^C-NMR (CDCl3 125 MHz): δ 173.69 (C31), 150.93 (C20), 109.36 (C29), 80.61(C3), 55.40 (C5), 50.35 (C9), 48.30 (C18), 48.01 (C19), 33.85(C32), 14.02 (C43).

## Discussion

In the present study, we aimed to investigate cytotoxicity of Fig latex, in this content, the in-vitro and *in-vivo* study were done, followed by column chromatography that was employed for purification of chloroform fraction. Afterwards, NMR and mass spectrophotometry were applied for compound identification. Interestingly, not only methanol fraction showed no cytotoxicity but also caused growth stimulation at 72 h. The growth stimulation of methanol fraction of *Ficus* family was reported previously by Kofi Annan (2008) (32). They reported this property could related to its antioxidant capacity (32). Both effects of Fig latex methanol fraction could be due to phenolic compounds that were identified in water fraction of Fig latex, despite phenolic compounds in water fraction. Tezcan *et al.*, (2016) showed that among four fractions of Fig latex including n-hexane, dichloromethane, ethanol, and water fraction only water fraction was cytotoxic on U-87 MG GBM cell lines (6). The possible reason for this difference can be the use of different cell lines and different molecular pathway. 

This is the first report of cytotoxicity of hydrophobic fractions. As we showed in the present study both employed hydrophobic fractions (including chloroform and n-hexane) of Fig latex showed the cytotoxic effect. The chloroform fraction was obviously more cytotoxic.

A study by Tezcan *et al*., (2016) reported no cytotoxicity by hydrophobic fractions of Fig latex (6). This discrepancy could be due to the low solubility of hydrophobic fractions in culture media and suitable solvents such as DMSO which results in low delivery of fractions to cells and lack of cytotoxicity. Phytochemical analysis related to the cytotoxic effect of Fig latex is limited to one study performed by Mechoulam *et al*. (2002). They reported the cytotoxic effect of Fig latex is because of 6-o-acyl-beta-d-glucosyl-beta-sitosterol, a responsible compound for Fig latex cytotoxic activity (33).

According to our phytochemical analysis, lupeol acetate and lupeol palmitate were present in the chloroform fraction. Also, the presence of lupeol acetate was previously reported by Oliveira *et al., *(20) but the isolation and identification of lupeol palmitae from Fig latex is reported for the first time. Oliveira *et al*. reported the presence of free fatty acids including palmitate in Fig latex but in our study, the fatty acid was not free and it was esterified with lupeol (20). Both compounds have triterpenoid structure with low solubility in water, approximately 0.02 µg/mL (21,34), thus do not dissolve in cell culture media. To improve solubility, we employed safe egg yolk phosphatidylcholine (EPC) to prepare liposome formulation and enhance delivery of hydrophobic fractions to cells. As we observed in our study, chloroform fraction was more cytotoxic with IC50 value of 0.219 and 0.748 mg/mL on HepG2 as well as NIH cell lines respectively. As mentioned above, the lupeol derivates were the major component in chloroform fraction; it seems that the cytotoxic effect is related to them. Several studies reported that lupeol causes cell death in cancerous cell more specifically (35) which was demonstrated in our study too, because the IC50 value for HepG2 cell was 3 times lower than that for NIH cell line. 

Moreover, chloroform fraction induces apoptosis in both HepG2 and NIH cell lines at IC50 concentration. The flow cytometry with Annexin V/ PI measurement, which is a well-known way for elucidation of cell death pathway, demonstrate presence of cells in early apoptosis phase (36). Both isolated compounds are lupeol derivates, it has been observed that lupeol interferes with various signaling pathway and stimulate apoptosis (37). It causes cell death in caspase 3 dependent pathway and inhibition of PI3K pathway (38). Previously the cytotoxicity and apoptosis induction of lupeol was observed in several types of cancers (39), therefore lupeol has already been considered as an anticancer chemo-preventive agent (40). It seems that the cytotoxicity and apoptosis induction of chloroform fraction are related to lupeol derivates which were isolated. 

For safety assay, the liposomal form of chloroform fraction and EPC were used. Administration of EPC (4.5 g/kg) as a vehicle of chloroform fraction caused no death or obvious histopathological changes on the liver which means the compound is completely safe. Although the liposomal form of the fraction was not lethal up to 2 g/kg, it caused 30% death at dose of 3 g/kg which classifies it as a safe product. According to phytochemical analysis and presence of lupeol derivates, reported LD50 for lupeol is more than 2g/kg (41) that is in line with our study. 

**Table 1:** **The dry weigh (g) of each fraction in 50 mL of Fig latex**

**Fraction**	**Weight in 50 mL of latex**
n-Hexan	5.987
Chloroform	2.277
Ethyl acetate	1.161
Methanol	2.91
Residue	2.277

**Table 2:** **Description of study groups, dose and type of administration**

**groups**	**Administration**
**a**	EPC 1.5 g/kg, i.p
**b**	EPC 3 g/kg, i.p
**c**	EPC 4.5 g/kg, i.p
**d**	Fraction 1 g/kg. i.p, Fraction: EPC (1:1.5)
**e**	Fraction 2 g/kg. i.p, Fraction: EPC (1:1.5)
**f**	Fraction 3 g/kg. i.p, Fraction: EPC (1:1.5)

a, b, and c: control groups; d, e, and f: test groups.

**Table 3:** IC50 values of four fractions of Fig latex on HepG2 and NIH3T3 cell lines using MTT assay

	**HepG2**			**NIH3T3**		
	**Time**			**Time**		
	24 h	48 h	72 h	24 h	48 h	72 h
	Not toxic	Not toxic	Not toxic	Not toxic	Not toxic	Not toxic
Ethyl-acetate	2.83 ± 0.14	2.7 ± 0.11	2.5 ± 0.21	3.8 ± 0.11	3.27 ± 0.27	3.04 ± 0.31
Chloroform	0.219 ± 0.01	0.21 ± 0.00	0.2 ± 0.02	0.748 ± 0.07	0.606 ± 0.12	0.332 ± 0.04
n-Hexane	2.51 ± 0.10	2.65 ± 0.13	2.67 ± 0.13	Not toxic	Not toxic	Not toxic

Values are reported in mg/mLand as Mean ± SEM.

**Table 4:** **The liver function test in animals treated with Chloroform fraction and its carrier EPC**

**Group**	**ALT (U/L)**	**AST (U/L)**	**ALP(U/L)**
Fraction 1 g/kg	13.96 ± 2.46	26.33 ± 9.87	76.27 ± 9.74
Fraction 2 g/kg	15.02 ± 5.34	30.19 ± 4.93	78.45 ± 13.89
Fraction 3 g/kg	17.71 ± 0.82	37.84 ± 4.93	95.26 ± 19.17
EPC 1.5 g/kg	19.012 ± 1.87	30.65 +1.23	76.22 ± 24.40
EPC 3 g/kg	15.52 ± 1.46	33.56 ± 4.11	87.76 ± 22.74
**EPC 4.5 g/kg**	15.908 ± 1.46	33.66 ± 1.23	89.96 ± 26.96

Results were shown as mean ± SD there is no statistically significant differences between groups.

## Conclusion

In Conclusion, the pharmacological activity of the chloroform fraction of Fig latex can be summarized as the following points: the chloroform fraction is considered as a safe fraction with LD50 > 3g/kg in mice. It has showed pro-apoptotic effect on cancerous cell line and the HepG2 cell line was three times more sensitive to apoptosis induction of chloroform fraction. Lupeol acetate and lupeol palmitate were the main compounds presented in chloroform fraction of Fig latex, which is related to cell cytotoxicity and apoptosis induction. Presence of the lupeol palmitat in Fig latex is reported for the first time. Further investigation is needed for characterizing the differences between the function of lupeol and its ester form in cancer. In this study, the liposome form of the fraction is used to solve hydrophobic fractions. The other forms of drug delivery such as PLA and PLGA nanoparticle might be useful for improving solubility of the hydrophobic fraction of Fig latex. 

## Funding

This study was supported by a grant (93-04-92-27752) from Vice Chancellor for Research, Tehran University of Medical Sciences, Tehran, Iran. 

## References

[1] Puppala S, Patel R, Yap KS, Patel J, Wah T, Snoddon A (2016). Hepatocellular carcinoma: modern image-guided therapies. Postgrad. Med. J..

[2] Mittal S, El-Serag HB (2013). Epidemiology of HCC: Consider the Population. Clin. Gastroenterol..

[3] Kanwal F, Hoang T, Kramer JR, Asch SM, Goetz MB, Zeringue A, Richardson P, El-Serag HB (2011). Increasing prevalence of HCC and cirrhosis in patients with chronic hepatitis C virus infection. Gastroenterology.

[4] Li Y, Martin RCG (2011). Herbal Medicine and Hepatocellular Carcinoma: Applications and Challenges. Evid.-Based Complementary Altern. Med..

[5] Chen SR, Qiu HC, Hu Y, Wang Y, Wang YT (2016). Herbal Medicine Offered as an Initiative Therapeutic Option for the Management of Hepatocellular Carcinoma. Phytother. Res..

[6] Tezcan G, Tunca B, Bekar A, Yalcin M, Sahin S, Budak F, Cecener G, Egeli U, Demir C, Guvenc, Yilmaz GG, Erkan LG, Malyer H, Taskapilioglu MO, Bilir TEA (2015). Ficus carica latex prevents invasion through induction of let-7d expression in GBM cell lines. Cell Mol. Neurobiol..

[7] Mawa S, Husain K, Jantan I (2013). Ficus carica L (Moraceae): phytochemistry, traditional uses and biological activities. Evid.-Based Complementary Altern. Med..

[8] Hemmatzadeh F, Fatemi A, Amini F (2003). Therapeutic effects of fig tree latex on bovine papillomatosis. J. Vet. Med. B.

[9] Koka S, Barik R, Joshi J, Jain S (2013). Effect of Ficus carica fruit extract on experimentally induced inflammation and nociception. J. Pharm. Pharm. Sci..

[10] Eteraf-Oskouei T, Allahyari S, Akbarzadeh-Atashkhosrow A, Delazar A, Pashaii M, Gan SH, Najafi M (2015). Methanolic Extract of Ficus carica Linn Leaves Exerts Antiangiogenesis Effects Based on the Rat Air Pouch Model of Inflammation. Evid.-Based Complementary Altern. Med..

[11] Mostafaie A, Mansouri K, Norooznezhad A-H, Mohammadi-Motlagh H-R (2011). Anti-angiogenic activity of Ficus carica latex extract on human umbilical vein endothelial cells. cells. Cell J (Yakhteh).

[12] Nagaty H, Rifaat M, Morsy T (1959). Trials of the effect on dog Ascaris in-vivo produced by the latex of Ficus carica and Papaya carica growing in Cairo gardens. Pathog. Glob. Health.

[13] Hashemi S, Abediankenari S, Ghasemi M, Azadbakht M, Yousefzadeh Y, Dehpour A (2011). The effect of fig tree latex (Ficus carica) on stomach cancer line. IRCMJ.

[14] Hashemi SA, Abediankenari S (2013). Suppressive Effect of Fig (Ficus carica) Latex on Esophageal Cancer Cell Proliferation. Acta Fac. Med. Naiss..

[15] Khodarahmi GA, Ghasemi N, Hassanzadeh F, Safaie M (2011). Cytotoxic effects of different extracts and latex of Ficus carica L. on HeLa cell line. Iran. J. Pharma.R..

[16] Chawla A, Kaur R, Sharma A (2012). Ficus carica Linn A review on its pharmacognostic, phytochemical and pharmacological aspects. Int. J. Pharm. Phytopharmacol. Res..

[17] Wang J, Wang X, Jiang S, Lin P, Zhang J, Lu Y, Wang Q, Xiong Z, Wu Y, Ren J, Yang H (2008). Cytotoxicity of fig fruit latex against human cancer cells. FCT.

[18] Conforti F, Menichini G, Zanfini L, Tundis R, Statti G, Provenzano E, Menichini F, Somma F, Alfano C (2012). Evaluation of phototoxic potential of aerial components of the fig tree against human melanoma. Cell Prolif..

[19] Rubnov S, Kashman Y, Rabinowitz R, Schlesinger M, Mechoulam R (2001). Suppressors of cancer cell proliferation from fig (Ficus c arica) resin: isolation and structure elucidation. J. Nat. Prod..

[20] Oliveira AP, Silva LsR, Andrade PB, Valentão Pc, Silva BM, Gonçalves RF, Pereira JA and Pinho PG (2010). Further insight into the latex metabolite profile of Ficus carica. J. Agric. Food Chem..

[21] Chairez-Ramirez MH, Sanchez-Burgos JA, Gomes C, Moreno-Jimenez MR, Gonzalez-Laredo RF, Bernad-Bernad MJ, Medina-Torres L, Ramírez-Mares MV, Gallegos-Infante, and Rocha-Guzmán NE (2015). Morphological and release characterization of nanoparticles formulated with poly (dl-lactide-co-glycolide) (PLGA) and lupeol: In-vitro permeability and modulator effect on NF-kappaB in Caco-2 cell system stimulated with TNF-alpha. FCT.

[22] Henry F, Danoux L, Pauly G, Charrouf Z (2005). A plant extract and its pharmaceutical and cosmetic use. Google Patents.

[23] Jain N, Gupta BP, Thakur N, Jain R, Banweer J, Jain DK, Jain S (2010). Phytosome: a novel drug delivery system for herbal medicine. IJPSDR.

[24] Shakeri A, Sahebkar A (2016). Phytosome: A Fatty Solution for Efficient Formulation of Phytopharmaceuticals. Recent Pat. Drug Deliv. Formul..

[25] Luz LEC, Kanunfre CC, Paludo KS, da Silva Justo A, Petry VK, Lemes BM, Barison A, Nepel A, Wang M, Avula B, Khan IK and Beltrame FL (2016). Cytotoxic biomonitored study of Euphorbia umbellata (Pax) Bruyns. J. Ethnopharmacol..

[26] Samy A, Elmowafy M, Arafa M, Kari O, Viitala T, Kassem A, Abu-Elyazid SK, Ibrahim HM, Yliperttula M (2014). Complement activation assay and in-vivo evaluation of silymarin loaded liver targeting liposome. J. Life Med..

[27] Khosroshahi ME, Hassannejad Z, Firouzi M, Arshi AR (2015). Nanoshell-mediated targeted photothermal therapy of HER2 human breast cancer cells using pulsed and continuous wave lasers: an in-vitro study. Lasers Med. Sci..

[28] Mohammadpour F, Ostad SN, Aliebrahimi S, Daman Z (2017). Anti-invasion Effects of Cannabinoids Agonist and Antagonist on Human Breast Cancer Stem Cells. Iran. J. Pharma. R..

[29] Guideline P-BT (2001). OECD guideline for the testing of chemicals. The Hershberger..

[30] Tavakoli S, Delnavazi M-R, Hadjiaghaee R, Jafari-Nodooshan S, Khalighi-Sigaroodi F, Akhbari M, Hadjiakhoondi A, Yassa N (2017). Bioactive coumarins from the roots and fruits of Ferulago trifida Boiss an endemic species to Iran. Nat. Prod. Res..

[31] Bodaghi-Namileh V, Sepand MR, Omidi A, Aghsami M, Seyednejad SA, Kasirzadeh S, Sabzevari O (2018). Acetyl-l-carnitine attenuates arsenic-induced liver injury by abrogation of mitochondrial dysfunction, inflammation, and apoptosis in rats. Environ. Toxicol. Pharmacol..

[32] Annan K, Houghton PJ (2008). Antibacterial, antioxidant and fibroblast growth stimulation of aqueous extracts of Ficus asperifolia Miq and Gossypium arboreum L wound-healing plants of Ghana. J Ethnopharmacol..

[33] Mechoulam R, Rubnov S, Kashman Y, Rabinowitz R, Schlesinger M (2002). Anti-proliferative 6-o-acyl-beta-d-glucosyl-beta-sitosterol compounds. Google Patents.

[34] Abdalrahim FAA, Abdul-Majid AM, Zhari I (2014). Preparation and characterization of nano liposomes of Orthosiphon stamineus ethanolic extract in soybean phospholipids. BMC Biotechnol..

[35] Namrata D, Bhavna D, Skand M, Yogeshwer S (2014). Lupeol Induced Apoptosis in Human Lung Cancer Cell Line: A Flow Cytometry Study. RJPPD.

[36] Badmus JA, Ekpo OE, Hussein AA, Meyer M, Hiss DC (2015). Antiproliferative and Apoptosis Induction Potential of the Methanolic Leaf Extract of Holarrhena floribunda (G Don). Evid.-Based Complementary Altern. Med..

[37] Abd Hamid H, Mutazah R, Yusoff MM, Abd Karim NA, Abdull Razis AF (2017). Comparative analysis of antioxidant and antiproliferative activities of Rhodomyrtus tomentosa extracts prepared with various solvents. FCT..

[38] Zhang L, Tu Y, He W, Peng Y, Qiu Z (2015). A novel mechanism of hepatocellular carcinoma cell apoptosis induced by lupeol via Brain-Derived Neurotrophic Factor Inhibition and Glycogen Synthase Kinase 3 beta reactivation. Eur. J. Pharmacol..

[39] Chaturvedi PK, Bhui K, Shukla Y (2008). Lupeol: Connotations for chemoprevention. Cancer Lett..

[40] Khatun M, Habib MR, Rabbi MA, Amin R, Islam MF, Nurujjaman M, Rezaul-Karim M, Habibur-Rahman M (2017). Antioxidant, cytotoxic and antineoplastic effects of Carissa carandas Linn. leaves. Exp. Toxicol. Pathol..

[41] National Center for Biotechnology Information PubChem Compound Database; CID = 259846.

